# Transcriptome assembly and differential gene expression of the invasive avian malaria parasite *Plasmodium relictum* in Hawaiʻi

**DOI:** 10.1002/ece3.7401

**Published:** 2021-03-27

**Authors:** Elin Videvall, Kristina L. Paxton, Michael G. Campana, Loren Cassin‐Sackett, Carter T. Atkinson, Robert C. Fleischer

**Affiliations:** ^1^ Center for Conservation Genomics Smithsonian Conservation Biology Institute National Zoological Park Washington DC USA; ^2^ Department of Biology University of Louisiana Lafayette LA USA; ^3^ U.S. Geological Survey Pacific Island Ecosystems Research Center Kilauea Field Station Hawai‘i National Park HI USA; ^4^ Present address: Hawai‘i Cooperative Studies Unit University of Hawai'i at Hilo Hawai‘i National Park HI USA

**Keywords:** *Chlorodrepanis*, gene expression, Hawaiʻi ʻamakihi, hemosporidia, *Plasmodium*, transcriptomics

## Abstract

The malaria parasite *Plasmodium relictum* (lineage GRW4) was introduced less than a century ago to the native avifauna of Hawaiʻi, where it has since caused major declines of endemic bird populations. One of the native bird species that is frequently infected with GRW4 is the Hawaiʻi ʻamakihi (*Chlorodrepanis virens*). To achieve a better understanding of the transcriptional activities of this virulent parasite, we performed a controlled challenge experiment of 15 ʻamakihi that were infected with GRW4. Blood samples containing malaria parasites were collected at two time points (intermediate and peak infection stages) from host individuals that were either experimentally infected by mosquitoes or inoculated with infected blood. We then used RNA sequencing to assemble a high‐quality blood transcriptome of *P. relictum* GRW4, allowing us to quantify parasite expression levels inside individual birds. We found few significant differences (one to two transcripts) in GRW4 expression levels between host infection stages and between inoculation methods. However, 36 transcripts showed differential expression levels among all host individuals, indicating a potential presence of host‐specific gene regulation across hosts. To reduce the extinction risk of the remaining native bird species in Hawaiʻi, genetic resources of the local *Plasmodium* lineage are needed to enable further molecular characterization of this parasite. Our newly built Hawaiian GRW4 transcriptome assembly, together with analyses of the parasite's transcriptional activities inside the blood of Hawaiʻi ʻamakihi, can provide us with important knowledge on how to combat this deadly avian disease in the future.

## Graphical abstract

In this study, we sequenced and built the first transcriptome assembly of the invasive Hawaiian malaria parasite, *Plasmodium relictum* lineage GRW4. Using a controlled infection experiment, we evaluated parasite gene expression levels during two infection time points in the blood of 15 native Hawaiʻi ʻamakihi (*Chlorodrepanis virens*).

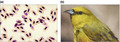

## 1 INTRODUCTION

**FIGURE 1:**
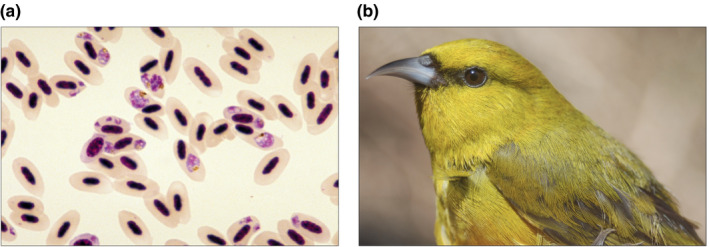
(a) Image of *Plasmodium relictum* GRW4 on a Giemsa‐stained blood smear seen through a microscope. Red blood cells are pictured, each containing an elongated nucleus in dark purple color. The pink round shapes within some of the cells constitute the parasites. Image by Carter T. Atkinson. (b) The host species, Hawaiʻi ʻamakihi (*Chlorodrepanis virens*). Photograph by Loren Cassin‐Sackett

Avian malaria is a debilitating introduced disease inflicting severe damage to the native avifauna of Hawaiʻi, in particular the unique radiation of Hawaiian honeycreepers (Jarvi et al., 2001; van Riper et al., 1986; Warner, 1968). Multiple endemic bird species have already gone extinct and most of the remaining honeycreepers are currently in population decline (Atkinson & LaPointe, 2009), with much of the endangerment attributable to avian malaria. Malaria is an infectious disease caused by single‐celled eukaryotic parasites in the genus *Plasmodium*, which are transmitted by mosquitoes. In 1826, the bird‐biting mosquito *Culex quinquefasciatus* was introduced to the Hawaiian islands with ships (Hardy, 1960), though it was not until the late 1930s that malaria parasites were discovered in the blood of native birds (van Riper et al., 1986). The parasites were identified as the broadly distributed mitochondrial lineage GRW4 of *Plasmodium relictum* (Figure 1a) (Beadell et al., 2006). Because the Hawaiian avifauna likely evolved for millions of years in the absence of malaria parasites (Fleischer et al., 1998; Lerner et al., 2011), many native bird species, and nearly all species of Hawaiian honeycreepers, do not possess much natural resistance or tolerance against the disease. As a result, Hawaiian honeycreepers often experience high levels of mortality when infected (e.g., Hawaiʻi ʻamakihi [*Chlorodrepanis virens*]: ~65%; ʻIʻiwi [*Drepanis coccinea*]: 90%) (Atkinson et al., 1995, 2000; van Riper et al., 1986), complicating conservation efforts to save these species from extinction.

Despite the urgency in understanding how *P. relictum* affect the endemic avifauna of Hawaiʻi, we know almost nothing of the parasite's transcriptional activities inside its hosts. Malaria parasite gene expression levels in birds have previously been evaluated in two species: *Plasmodium ashfordi* and *Plasmodium homocircumflexum* (Garcia‐Longoria et al., 2020; Videvall et al., 2017). The first study found that *P. ashfordi* gene expression did not differ between peak and decreasing parasitemia stages in Eurasian siskins; instead, 28 transcripts showed differential expression depending on which host individual the parasites infected (Videvall et al., 2017). Similarly, the study evaluating *P. homocircumflexum* found transcriptional differences between hosts; however, this approach evaluated differences across bird species (crossbills and starlings) (Garcia‐Longoria et al., 2020). While these previous results provide valuable information, the sample sizes were limited to three–four individuals (and two time‐points for *P. ashfordi*) and thus require further investigation. In addition, European birds with evolved resistance to malaria are not able to provide accurate estimates of how *P. relictum* behaves in the blood of native bird species of Hawaiʻi, which have evolved without the parasite.

In this study, we sequenced and built the first transcriptome assembly of *P*. *relictum* (lineage GRW4). Using a controlled infection experiment, we evaluated parasite gene expression levels during two time points in the blood of 15 native, high‐elevation Hawaiʻi ʻamakihi. We specifically aimed to evaluate whether parasite gene expression differs (a) between intermediate and peak infection stages, (b) between birds that survived and birds that died from malaria, (c) between a mosquito inoculation method and blood injection method, and finally (d) among different host individuals.

## 2 METHODS

### 2.1 Experimental design

We captured 20 individuals of Hawaiʻi ʻamakihi (Figure 1b) in August and September of 2015 on the island of Hawaiʻi in the Upper Waiākea Forest Reserve. This high‐elevation region is predominantly malaria free where birds are unlikely to encounter malaria parasites in the wild. The birds were kept in individual cages in a mosquito‐proof aviary, subjected to natural light, and provided a diet of nectar, fruit, and vegetables. Prior to the experiment, all birds were screened with nested PCR (Lapointe et al., 2016), ELISA (Woodworth et al., 2005), and microscopy to ensure no individual carried hemosporidian infection. The *P. relictum* GRW4 isolate KV115 was used, originally obtained from a wild ʻapapane (*Himatione sanguinea*) at Kilauea Crater in Hawaiʻi Volcanoes National Park in 1992. It was passaged once in a canary (*Serinus canaria*) prior to being glycerolized, then frozen and stored in liquid nitrogen. The same isolate has been used in previous experimental studies (Atkinson et al., 2000, 2013). Prior to the experiment in this study, the isolate was thawed, deglycerolized, and passaged in canaries an additional four times.

Birds were acclimated for a minimum of four weeks before being randomly assigned to one of three treatment groups: control, inoculation by mosquitoes, or inoculation by blood injection. Ten birds were infected through exposure overnight to the bite of a single infected *Culex quinquefasciatus* (mosquito inoculation group). The mosquitoes had been infected by a single canary that was inoculated with *P. relictum* GRW4. Five birds were experimentally infected by subinoculation in their pectoral muscle with 150 μl of infected blood solution sourced from the same canary individual that infected the mosquitoes (blood inoculation group). The last five birds were exposed overnight to the bite of a single uninfected *C*. *quinquefasciatus* mosquito (control group). The control birds were not included in this study because they harbored no parasites to sequence. Results on how the avian hosts responded (physiologically and transcriptionally) to malaria will be published in a future companion paper (Kristina L. Paxton, University of Hawaiʻi at Hilo, written communication, 2020).

Experimental procedures were approved by the Smithsonian National Zoological Park Institutional Animal Care and Use Committee (NZP‐IACUC Proposal #15‐18). Other permits included the following: U.S. Fish and Wildlife Service Migratory Bird Scientific Collection Permit (MB67895B), U.S. Department of the Interior Bird Banding Laboratory (permit #21144), Hawaiʻi State Protected Wildlife Research Permit (WL 17–08), and Hawaiʻi State Access and Forest Reserve Special Use Permit.

### 2.2 Parasitemia quantification

We measured intensity of parasitemia using a quantitative PCR (qPCR) assay with a hydrolysis probe (Kristina L. Paxton, University of Hawaiʻi at Hilo, written communication, 2020) to test whole blood samples collected every 5 days postinoculation. Previously published GRW4‐specific primers (Zehtindjiev et al., 2008) that target cytochrome *b* were used. We designed a fluorescent probe (5′‐5TEX615‐GCT‐TTT‐GGT‐GCA‐AGA‐GAG‐TAT‐TCA‐GT‐31AbRQsp‐3′) with high specificity to the GRW4 target sequence. Genomic DNA was extracted from blood samples using the DNeasy tissue extraction kit (Qiagen), quantified using a Qubit fluorometer (Invitrogen), and normalized to a starting concentration of 2 ng/µl. Reactions were run in a final volume of 20 µl, including 10 µl of iTaq Universal Probe Supermix (Bio‐Rad Laboratories), 2 µl of DNA, 1.5 µl of each primer (10 µM), 0.5 µl of probe (10 µM), and 4.5 µl of PCR water. We performed the qPCR in a C1000 Touch Thermocycler with a CFX96 Real‐time System (Bio‐Rad Laboratories) with the following thermal profile: 5 min at 95°C followed by 40 cycles of 5 s at 95°C and 30 s at 59°C. Samples were run in triplicate, along with a negative and positive control, and a serial dilution of a gBlocks Gene Fragment (IDT) containing the target sequence and a starting copy number of 5.8 × 10^6^. The *C*_T_ value (cycle threshold) of each sample was calculated as the mean of the three replicates and only included if the difference between them was <1 *C*_T_ value. We estimated relative parasitemia of each sample based on the serial dilution of the gBlock Gene Fragment included in each run and converted *C*_T_ values to SQ values (Starting Quantity). The average amplification efficiency of all runs was 92.8 ± 1.8%.

At each sampling period, we also collected samples for RNA sequencing (30 μl whole blood in 210 μl of RNAlater) that were stored for 24 hr at 0°C and then at −20°C until RNA extraction. Blood smears were prepared, air‐dried, fixed with methanol, and stained with 6% buffered Giemsa for 1 hr. They were then examined by microscopy to determine the proportion of asexual and sexual parasites in 100–200 infected erythrocytes. Blood smear examination was performed without prior knowledge of experimental group.

Based on prior experimental studies (Atkinson et al., 2000, 2013), ʻamakihi were classified as fatalities when their parasitemia levels exceeded 20%, food consumption fell below 5 ml of nectar over the prior 24‐hr period, and individuals appeared moribund. Five birds were classified as fatalities, removed from the experiment, and treated with oral chloroquine (10 mg/kg) to reduce risk of dying without intervention. Despite these efforts, four of these five birds died within a few weeks of chloroquine treatment.

### 2.3 RNA extraction and sequencing

RNAlater was separated from blood by centrifugation, and RNA from approximately 20 μl of packed red blood cells was subsequently extracted using Dynabeads mRNA Direct Kit (Invitrogen), a poly‐A tail binding bead‐based approach that captures mRNA. We converted mRNA to first‐ and second‐strand cDNA using SuperScript III Reverse Transcriptase (RT‐PCR; Invitrogen) with random hexamer primers, and NEBNext mRNA Second Strand Synthesis Module (New England Biolabs). Samples were normalized to a starting concentration of 0.2 ng/μl, and individual libraries were prepared using Nextera XT Library Prep Kit (Illumina), which fragments cDNA and tags each individual sample with a unique combination of two barcoded Illumina primers. The cDNA libraries were quantified on an Agilent 2100 Bioanalyzer High Sensitivity DNA chip, pooled in equimolar ratios, and size‐selected using a Pippin Prep (Sage Science). Paired‐end, 150‐base pair (bp) sequencing was performed on an Illumina Hiseq 2500 (Johns Hopkins Genetic Resources Core Facility).

We sequenced mRNA from a total of 34 infected blood samples collected from 15 birds. Of these, 30 samples were derived from all hosts during two infection stages: intermediate stage (sampling period in between day 0 and peak infection) and peak stage (peak parasitemia for survivors and the sample closest to removal from experiment for fatalities). Four additional samples from hosts collected 5 days before the intermediate infection were also sequenced; however, those samples did not have high enough parasitemia for gene expression analyses so were only included in the assembly‐building process.

### 2.4 Transcriptome assembly

We assembled the transcriptome of *P. relictum* GRW4 using Trinity (v. 2.6.6) (Grabherr et al., 2011) based on sequences from all 34 infected samples. First, high‐quality sequence read pairs (479.8 million) were mapped using STAR (v. 2.5.4b) (Dobin et al., 2013) to the genome of *P. relictum* DONANA05 (SGS1‐like) (Böhme et al., 2018) downloaded from PlasmoDB (v. 39) (Aurrecoechea et al., 2009). Parameters in STAR were set to be optimized for *Plasmodium* parasites (Baruzzo et al., 2017), slightly modified to fit our data (Table S2). The GRW4 transcriptome was subsequently de novo‐assembled with Trinity's genome‐guided approach using the STAR‐mapped reads to help guide the assembly process. Trinity's genome‐guided transcriptome assembly method uses aligned reads partitioned according to locus, followed by de novo assembly at each locus (Haas, 2020). This method is distinct from typical genome‐guided approaches because transcripts are constructed de novo and the provided genome is only being used as a substrate for grouping overlapping reads into clusters (Haas, 2020). Maximum intron size was set to 4,000 based on *Plasmodium* genomes (Aurrecoechea et al., 2009) and minimum contig length to the default of 200 bp. Next, we clustered similar isoforms into transcripts using CD‐HIT‐EST (v. 4.6) (Li & Godzik, 2006) with a 90% similarity threshold. The transcripts were subsequently blasted against UniProtKB/TrEMBL with blastx+ (v.2.9.0) (Camacho et al., 2009). Based on this blast search, 12,475 contigs (99.9%) gave significant matches (*e*‐value < 1e‐6) to *Plasmodium* and 17 contigs (0.1%) to organisms other than *Plasmodium* (e.g., bacteria, nematodes). The 17 non‐*Plasmodium* contigs were removed, and the filtered transcripts were again blasted but this time with blastn against the newly realigned genome of Hawaiʻi ʻamakihi (Callicrate et al., 2014; Campana et al., 2020). This search (*e*‐value < 1e‐10) resulted in 22 contigs matching potential avian rRNA sequences, which were all removed from the assembly. We subsequently used TransDecoder (v. 5.5.0) (Grabherr et al., 2011) to identify open reading frames and blastn against *P. relictum* DONANA05 (SGS1‐like) coding sequences for gene annotation.

### 2.5 Differential expression analyses

Sequence reads from all infected samples were first mapped individually using HiSat2 (v. 2.1.0) (Kim et al., 2019) without soft clipping against the genome of Hawaiʻi ʻamakihi (Campana et al., 2020), to remove the majority of bird‐derived sequences. We then extracted only the reads where both reads in a pair failed to align against the bird reference. These unmapped read pairs were subsequently mapped against the newly built transcriptome assembly of *P*. *relictum* GRW4 without soft clipping and spliced alignment (because transcriptome assemblies do not have splice junctions). The reads with alignments to the GRW4 transcriptome were then filtered with SAMtools (v. 1.9) (Li et al., 2009), retaining only reads with a mapping quality of >30. Next, HTSeq (v. 0.11.1) (Anders et al., 2015) was used to count the number of high‐quality mapped reads against GRW4 transcripts with mode set to “intersection‐nonempty”. To evaluate the percentages of parasite sequences in whole blood, we performed an additional read mapping procedure using HiSat2 against the GRW4 transcriptome, but this time we used the full set of unfiltered sequence reads (including host‐derived sequences). The proportion of total reads mapping against GRW4 showed a strong correlation with estimated parasitemia (Pearson's correlation test: *r* = .70, *p* = 4.52e‐06; see Figure S1), meaning that parasitemia intensity was a good predictor of *P. relictum* sequence depth.

Two samples during peak infection and 12 samples during intermediate infection stage had insufficient parasitemia (SQ values <50) and low mapping percentages (0.01%–0.03% of total reads) and could therefore not be used in gene expression analyses. Two birds (R71 and G32) were after the infection experiment found to harbor a secondary suspected aspergillosis infection and therefore excluded from the companion host gene expression study (Kristina L. Paxton, University of Hawaiʻi at Hilo, written communication, 2020). However, we retained the RNA‐seq samples from these two birds in this *Plasmodium* study, because we expect that aspergillosis in the respiratory tract is unlikely to have major effects on the transcription of *P. relictum* inside red blood cells (if the diseases occurred simultaneously). In addition, these birds produced a total of *n* = 3 samples with high parasitemia, which were deemed too valuable to simply exclude from a study already limited by sample size. In total, *n* = 20 samples had sufficiently high parasitemia to qualify for downstream expression analyses (SQ: 212–127,443); these originated from 13 individual hosts during the peak infection stage and seven individual hosts during the intermediate stage (Table S1).

We analyzed differential gene expression in R (v. 3.6.2) (R Core Team, 2019) using DESeq2 (v. 1.26.0) (Love et al., 2014). In DESeq2, read counts were normalized against library size according to the manual. This normalization procedure effectively enables direct comparisons between samples of different parasitemia levels (while allowing for contrasts between the two time points), because it controls for the number of reads that are mapped to the transcriptome. Prior to testing for differential expression, transcripts were filtered to include only those with a normalized expression count >10 across samples. We included the following variables in our DESeq2 model: host individual, inoculation method (mosquito/blood injection), mortality outcome (fatality/survivor), and stage of infection (peak/intermediate). This approach allowed us to extract and evaluate the four different variables separately while simultaneously controlling for the effects of the other factors. We tested for differential parasite transcript expression: (a) between peak and intermediate infection stages, (b) between fatalities and survivors, and (c) between mosquito‐infected and blood‐inoculated hosts. To evaluate (d) transcriptional differences among host individuals, we created a subset of the data that included all paired samples with parasite expression data at both time points of the infection (intermediate and peak; *n* = 7 hosts). Parasite expression differences across hosts were then tested using the likelihood ratio test (LRT) while controlling for the stage of infection. Inoculation method was not possible to include in the LRT model because only two of the host individuals with paired data had been subjected to the blood injection method (GR38 and GR39). *p*‐values were corrected to *q*‐values in all tests using the Benjamini and Hochberg false discovery rate, and transcripts were considered significantly differentially expressed using the default DESeq2 threshold of *q* < 0.1. Regularized logarithm transformation of data (rlog) was performed to remove dependence of the variance on the mean and used for heatmap data visualizations and principal component analysis (PCA) without any prior bias. Rlog is recommended over variance stabilizing transformation when library sizes differ between samples (Love et al., 2014), which they do in these types of data with large variation in parasitemia. Plots were made in R using ggplot2 (v. 3.2.1) (Wickham, 2011).

## 3 RESULTS

### 3.1 The blood transcriptome of *Plasmodium relictum* GRW4

**FIGURE 2:**
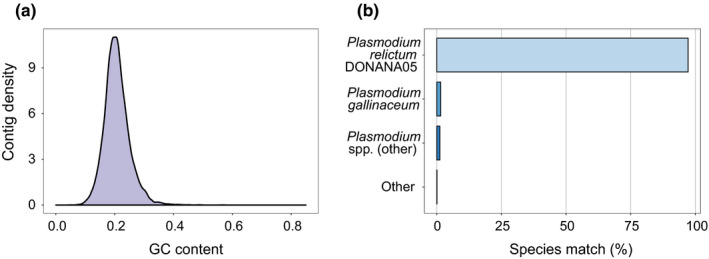
(a) Density curve of the *Plasmodium relictum* GRW4 transcriptome GC content (mean = 21.31%). (b) Proportion of GRW4 transcripts with the best blast match to species in the EBI TrEMBL protein database

The filtered *P. relictum* GRW4 transcriptome assembly comprised 15,594 contigs with a combined length of 11,261,992 bp. A majority of these contigs contained open reading frames (12,901). Like other avian *Plasmodium* transcriptomes, the assembly consisted of both longer and fragmented transcripts due to low parasite read coverage, yet it was of comparably high quality (mean transcript length = 722.2 bp; Table 1). The longest assembled contig (23,694 bp) matched the *Plasmodium* guanylyl cyclase gene. Transcriptome GC content was 21.31% (Figure 2a), similar to other avian *Plasmodium* transcriptomes (Table 1). Sequence similarity searches of *P. relictum* GRW4 transcripts against the EBI TrEMBL protein database showed that almost all contigs with significant hits matched the two available bird *Plasmodium* genomes (*n* = 12,328; 98.7%; Figure 2b). The remaining transcripts matched different mammalian *Plasmodium* species (*n* = 146; 1.2%) and other non‐*Plasmodium* microbes (*n* = 17; 0.1%). Of the 146 transcript matching mammalian malaria parasites, most resulted in matches against the human‐infecting species *Plasmodium falciparum* (*n* = 46; 0.4% overall) and *Plasmodium ovale* (*n* = 26; 0.2% overall).

**TABLE 1:** Assembly statistics of avian *Plasmodium* transcriptomes

Species	Transcriptome build	Nr contigs	Transcript GC (%)	Size (bp)	Mean transcript length (bp)	Reference
*Plasmodium relictum* GRW4	De novo assembly	15,594	21.31	11,261,992	722.2	This study
*Plasmodium ashfordi* GRW2	De novo assembly	11,954	21.22	9,010,380	930.8	Videvall et al. (2017)
*Plasmodium delichoni* COLL6	De novo assembly	12,048	23.93	5,680,962	471.5	Weinberg et al. (2019)
*Plasmodium homocircumflexum* COLL4	De novo assembly	21,612	21.64	17,175,763	794.7	Weinberg et al. (2019)
*Plasmodium homocircumflexum* COLL4	De novo assembly^a^	15,597	15.97	7,090,152	454.6	Garcia‐Longoria et al. (2020)
*Plasmodium gallinaceum*	De novo assembly^b^	32,549	28.42	22,074,210	678.2	Lauron et al. (2015)
*Plasmodium gallinaceum* 8A	Inferred from genome	5,439	21.21	11,238,032	2,066.0	Böhme et al. (2018)
*Plasmodium relictum* DONANA05 (SGS1‐like)	Inferred from genome	5,306	21.60	11,003,271	2,073.7	Böhme et al. (2018)

^a^ Assembly was cut off at 23% GC, so the associated numbers are not representative.

^b^ Assembly contains predominantly host transcripts, so the associated numbers are not representative.

### 3.2 Parasite gene expression does not differ between infection stages

**FIGURE 3:**
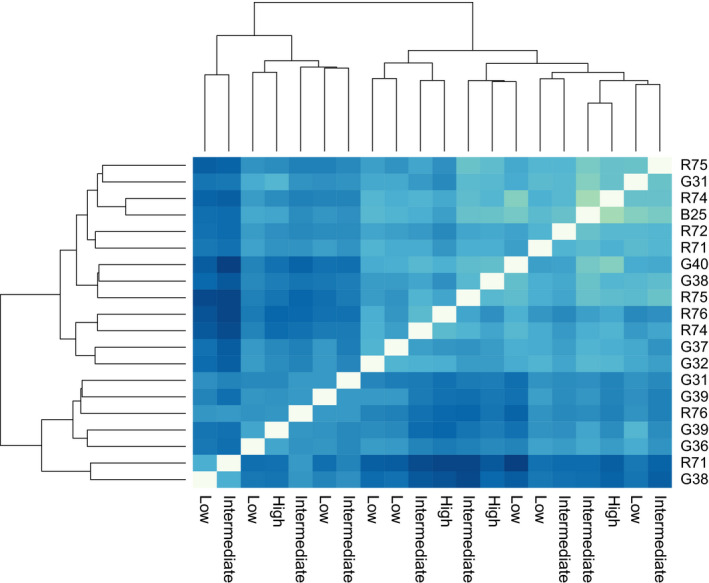
No clustering of transcriptomes based on parasitemia intensity. Euclidian distance heatmap together with dendrogram show that *Plasmodium relictum* gene expression patterns do not cluster based on parasitemia levels (here denoted as low, intermediate, high). Right side of the graph lists host IDs in the same sample order as the parasitemia levels given at the bottom of the graph. Darker colors indicate greater distance between samples, and white boxes denote identical samples

We quantified expression levels of all *P. relictum* transcripts in samples containing sufficient numbers of parasites (>200 SQ values; *n* = 20). The most highly expressed transcripts originated from genes previously documented as having the highest expression levels in other *Plasmodium* transcriptomes (Kim et al., 2017), for example, elongation factor 1‐alpha and 2, histone H4 and H2A, heat shock protein 70, and alpha tubulin 1 (Table S3). Evaluation of total transcriptome expression showed no clustering of samples based on similarity of parasitemia intensities (Figure 3), demonstrating the read normalization method removed potential biases associated with sequencing depth and parasite load.

**FIGURE 4:**
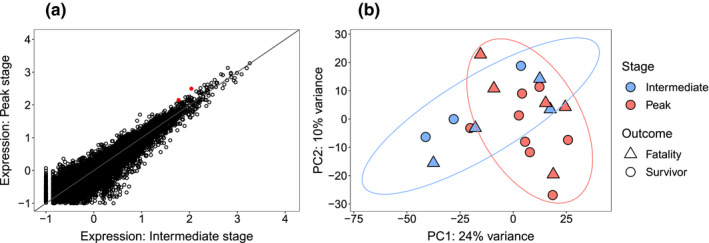
Few transcriptional differences in *Plasmodium relictum* between intermediate and peak host infection stages. (a) Differential gene expression analyses identified two upregulated *P. relictum* transcripts during the peak stage (red points). (b) PCA of *P. relictum* transcriptomes show samples from the two infection stages largely overlapping in gene expression. Ellipses denote the 90% confidence intervals

Comparing the two time stages of infection (peak and intermediate) also showed few differences in parasite transcript expression, with the exception of two transcripts upregulated during the peak infection stage (Figure 4). These transcripts belonged to a conserved *Plasmodium* gene with unknown function (PRELSG_0814200; Wald statistic = 3.9, *q* = 0.06) and a gene coding for DNA‐directed RNA polymerases I, II, and III (PRELSG_1105700; Wald statistic = 3.8, *q* = 0.06). We further found no differences in transcript expression between parasites in hosts that were classified as fatalities compared to parasites in hosts that survived the disease. Testing the effect of inoculation method (mosquito vs. blood injection) resulted in one significant transcript coding for gamete antigen 27/25 (PRELSG_0014900; Wald statistic = −4.1, *q* = 0.04), which had slightly higher expression in the parasites that had been injected with blood inoculation at the beginning of the experiment.

### 3.3 Parasite gene expression differs among host individuals

**FIGURE 5:**
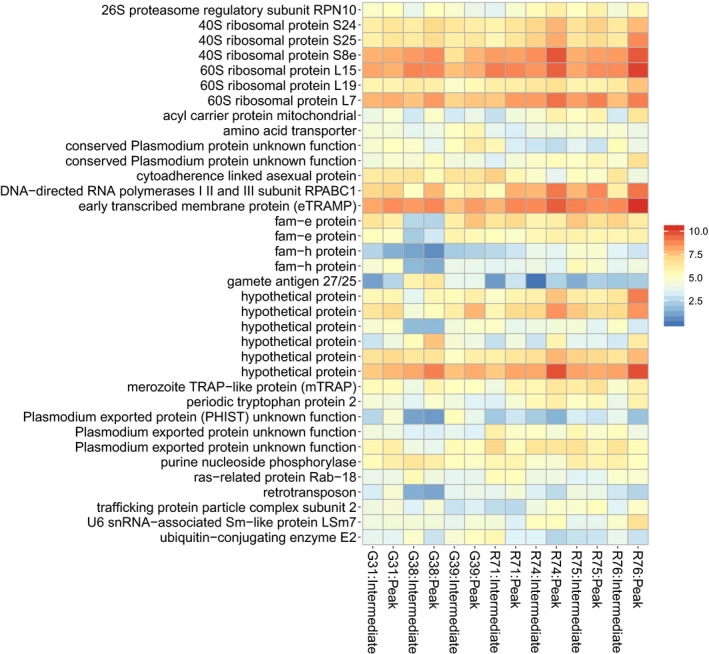
Expression levels of 36 *Plasmodium relictum* transcripts that were significantly differentially expressed in one or several host individuals (likelihood ratio test, controlled for time and parasitemia). The *y*‐axis shows the transcripts' protein products and the *x*‐axis depicts host individual:infection stage. Warmer colors indicate higher expression levels (rlog‐transformed transcript expression values). Transcript IDs can be found in Table S4

Seven birds had sufficiently high parasitemia levels during both infection time points (intermediate and peak) to allow for parasite gene expression analyses among host individuals while controlling for time (Figure S2). This analysis resulted in 36 *P. relictum* transcripts showing significantly different expression levels in one or several host individuals (Figure 5; Table S4). The most significant parasite transcripts showing expression differences were those coding for fam‐e and fam‐h proteins. Other highly significant transcripts included ribosomal proteins and conserved *Plasmodium* proteins with unknown function (Figure 5). Finally, we examined parasite developmental stages under a microscope and found no differences in the proportion of gametocytes across host individuals or host infection stages (mean gametocyte proportion: 1.7% peak infection and 6.2% intermediate infection; ANOVA Bird ID: *F* = 0.92, *p* = .56; stage: *F* = 4.18, *p* = .11).

## 4 DISCUSSION

We sequenced and assembled the blood stage transcriptome of the malaria parasite transmitted in the Hawaiian Islands, *P. relictum* GRW4, from experimentally infected Hawaiʻi ʻamakihi. The assembly is similar to other de novo assembled avian *Plasmodium* transcriptomes, including a GC content of 21.31% and a total size of ~11 Mbp. Unsurprisingly, almost all GRW4 transcripts (98.7%) matched most closely to the genome of the sister lineage *P. relictum* DONANA05 (Böhme et al., 2018).

The controlled infection experiment allowed us to evaluate gene expression of GRW4 in relation to host infection stage, mortality outcome, inoculation method, and host individual. We found almost no differences in parasite gene expression between peak and intermediate parasitemia infection stages as only two transcripts passed the significance threshold. Another study comparing time points during *P. ashfordi* infection in blood, similarly found no parasite expression differences between host parasitemia stages (Videvall et al., 2017). The lack of detectable differences may be due to (a) the fact that *Plasmodium* parasite expression patterns are usually associated with their developmental stage, and parasite populations in the blood of hosts are expected to be asynchronous (Lee et al., 2018). They are therefore likely to exhibit large variation in expression levels, and this averaging effect could potentially mask transcriptional differences across time points. (b) Another reason could be that the infection stages in our study were relatively close to each other in time to discern subtle differences, with peak infection taking place 5–10 days after the intermediate stage. The host immune response had not yet managed to suppress the parasite load at these stages of infection, as the intensity of parasitemia was at its highest during peak infection. However, this explanation does not apply to the *P. ashfordi* study (Videvall et al., 2017), which evaluated later time points (peak and decreasing parasitemia stages). (c) Low statistical power based on shallow parasite sequencing depth is also likely to explain part of why we find so few differences between time points, resulting from the fact that the vast majority of sequence reads originate from the host. Avian *Plasmodium* genomes are tiny in comparison to the genomes of their hosts, and because blood samples from birds predominantly contain nucleated host cells with high globin gene expression (Videvall, 2019), hemosporidian genomes and transcriptomes sequenced from birds unfortunately suffer from relatively low coverage.

Our RNA‐sequencing approach did, however, provide enough coverage to identify 36 transcripts that were significantly differentially expressed among seven host individuals. These differences could not be explained by the fact that some host individuals later died from the infection, because fatalities and survivors showed no differences in parasite gene expression. Besides a single transcript, there were also no differences in parasite expression between mosquito‐infected and blood‐inoculated individuals. The aforementioned study evaluating gene expression in *P. ashfordi* found similar results, with parasite transcripts differentially expressed across host individuals (Videvall et al., 2017), although the identified transcripts with known protein function differed from our results. Another study investigating gene expression of the GRW4 sister lineage, *P. relictum* SGS1, found large differences across developmental stages of SGS1 (Sekar et al., 2020), though this was evaluated in mosquitoes where parasites undergo several distinct stages in a successive time‐dependent manner. Different parasite stages are unlikely to explain our results given we found no differences in gametocytes identified in blood smears across host individuals or between host infection stages. It is possible that GRW4 is regulating the expression of certain genes to better respond to different host individuals; however, further studies are needed to evaluate the precise mechanism behind this pattern.

The most significant transcripts showing expression differences among host individuals belonged to the multigene families fam‐e and fam‐h. These subtelomeric gene families are not present in laveranian *Plasmodium* species but are expanded in avian *Plasmodium* genomes: fam‐e comprises four gene copies and fam‐h 49 gene copies in *P. relictum* (Böhme et al., 2018). Fam‐e has been discovered in the genome of *Plasmodium vivax* (Carlton et al., 2008), while fam‐h is believed to be specific to avian *Plasmodium* (Böhme et al., 2018). They appear related to the *P. falciparum* protein families RAD and PHIST, which bind the virulence factor PfEMP1 and remodel host erythrocytes (Oberli et al., 2014; Warncke et al., 2016). Several studies have found differential gene expression of PHIST during the *P. falciparum* life cycle and among different parasite isolates (Eksi et al., 2005; Rovira‐Graells et al., 2012). Interestingly, we also found one retrotransposon with differential expression levels among host individuals. Transposable elements are not present in mammalian *Plasmodium*, but they have been found in avian *Plasmodium* genomes (Böhme et al., 2018). It has been suggested that transposable elements like retrotransposons were present in genomes of ancestral apicomplexa and subsequently lost (Roy & Penny, 2007). The transcript in our study matches the intact *Plasmodium gallinaceum* Ty3/Gypsy LTR retrotransposon (PGAL8A_00410600), which has a continuous open reading frame. Because almost nothing is known about this particular retrotransposon, it is difficult to speculate why it is differentially expressed in *P. relictum*; however, we note that studies of *Entamoeba* have found differential transposon expression between strains (Macfarlane & Singh, 2006).

In conclusion, our results enable an improved understanding of the transcriptional activities of malaria parasites in birds, and the assembled transcriptome of *P. relictum* GRW4 will become a valuable genetic resource in the long‐term quest to better characterize the biology and evolution of this invasive *Plasmodium* lineage.

## CONFLICT OF INTEREST

The authors declare that they have no conflicts of interest.

## AUTHOR CONTRIBUTIONS

**Elin Videvall:** Data curation (lead); Formal analysis (lead); Validation (lead); Visualization (lead); Writing‐original draft (lead); Writing‐review & editing (lead). **Kristina Paxton:** Conceptualization (equal); Data curation (equal); Funding acquisition (equal); Investigation (lead); Methodology (lead); Project administration (lead); Resources (supporting); Writing‐review & editing (supporting). **Michael G. Campana:** Data curation (equal); Supervision (equal); Writing‐original draft (supporting); Writing‐review & editing (supporting). **Loren Cassin‐Sackett:** Methodology (supporting); Writing‐review & editing (supporting). **Carter Atkinson:** Conceptualization (supporting); Investigation (supporting); Methodology (supporting); Resources (supporting); Writing‐review & editing (supporting). **Robert C Fleischer:** Conceptualization (equal); Funding acquisition (lead); Project administration (equal); Resources (lead); Supervision (lead); Writing‐original draft (supporting); Writing‐review & editing (supporting).

## Supporting information

Fig S1‐S2

Table S1‐S4

## Data Availability

Supporting information will be made available online. Sequences have been uploaded to the Sequence Read Archive (SRA) at NCBI under accession number: PRJNA690103. The *P. relictum* GRW4 transcriptome assembly and the R code used in the analyses are available on FigShare (https://doi.org/10.25573/data.13611386). The assembly has additionally been deposited in the MalAvi database (http://mbio‐serv2.mbioekol.lu.se/Malavi/).

## References

[ece37401-bib-0001] Anders, S. , Pyl, P. T. , & Huber, W. (2015). HTSeq‐a Python framework to work with high‐throughput sequencing data. Bioinformatics, 31(2), 166–169. 10.1093/bioinformatics/btu638 25260700 PMC4287950

[ece37401-bib-0002] Atkinson, C. T. , Dusek, R. J. , Woods, K. L. , & Iko, W. M. (2000). Pathogenicity of Avian malaria in experimentally‐ infected Hawaii Amakihi. Journal of Wildlife Diseases, 36(2), 197–204. 10.7589/0090-3558-36.2.197 10813599

[ece37401-bib-0003] Atkinson, C. T. , & LaPointe, D. A. (2009). Introduced Avian diseases, climate change, and the future of Hawaiian honeycreepers. Journal of Avian Medicine and Surgery, 23(1), 53–63. 10.1647/2008-059.1 19530408

[ece37401-bib-0004] Atkinson, C. T. , Saili, K. S. , Utzurrum, R. B. , & Jarvi, S. I. (2013). Experimental evidence for evolved tolerance to avian malaria in a wild population of low elevation Hawai’i ’’i (*Hemignathus virens*). EcoHealth, 10(4), 366–375. 10.1007/s10393-013-0899-2 24430825

[ece37401-bib-0005] Atkinson, C. T. , Woods, K. L. , Dusek, R. J. , Sileo, L. S. , & Iko, W. M. (1995). Wildlife disease and conservation in Hawaii: Pathogenicity of avian malaria (*Plasmodium relictum*) in experimentally infected Iiwi (*Vestiaria coccinea*). Parasitology, 111(S1), S59–S69. 10.1017/S003118200007582X 8632925

[ece37401-bib-0006] Aurrecoechea, C. , Brestelli, J. , Brunk, B. P. , Dommer, J. , Fischer, S. , Gajria, B. , Gao, X. , Gingle, A. , Grant, G. , Harb, O. S. , Heiges, M. , Innamorato, F. , Iodice, J. , Kissinger, J. C. , Kraemer, E. , Li, W. , Miller, J. A. , Nayak, V. , Pennington, C. , … Wang, H. (2009). PlasmoDB: A functional genomic database for malaria parasites. Nucleic Acids Research, 37, 539–543. 10.1093/nar/gkn814 PMC2686598 18957442

[ece37401-bib-0007] Baruzzo, G. , Hayer, K. E. , Kim, E. J. , Di Camillo, B. , Fitzgerald, G. A. , & Grant, G. R. (2017). Simulation‐based comprehensive benchmarking of RNA‐seq aligners. Nature Methods, 14(2), 135–139. 10.1038/nmeth.4106 27941783 PMC5792058

[ece37401-bib-0008] Beadell, J. S. , Ishtiaq, F. , Covas, R. , Melo, M. , Warren, B. H. , Atkinson, C. T. , Bensch, S. , Graves, G. R. , Jhala, Y. V. , Peirce, M. A. , Rahmani, A. R. , Fonseca, D. M. , & Fleischer, R. C. (2006). Global phylogeographic limits of Hawaii’s avian malaria. Proceedings of the Royal Society B: Biological Sciences, 273(1604), 2935–2944. 10.1098/rspb.2006.3671 PMC1639517 17015360

[ece37401-bib-0009] Böhme, U. , Otto, T. D. , Cotton, J. A. , Steinbiss, S. , Sanders, M. , Oyola, S. O. , Nicot, A. , Gandon, S. , Patra, K. P. , Herd, C. , Bushell, E. , Modrzynska, K. K. , Billker, O. , Vinetz, J. M. , Rivero, A. , Newbold, C. I. , & Berriman, M. (2018). Complete avian malaria parasite genomes reveal features associated with lineage‐specific evolution in birds and mammals. Genome Research, 28(4), 547–560. 10.1101/gr.218123.116 29500236 PMC5880244

[ece37401-bib-0010] Callicrate, T. , Dikow, R. , Thomas, J. W. , Mullikin, J. C. , Jarvis, E. D. , & Fleischer, R. C. (2014). Genomic resources for the endangered Hawaiian honeycreepers. BMC Genomics, 15(1), 1098. 10.1186/1471-2164-15-1098 25496081 PMC4300047

[ece37401-bib-0011] Camacho, C. , Coulouris, G. , Avagyan, V. , Ma, N. , Papadopoulos, J. , Bealer, K. , & Madden, T. L. (2009). BLAST+: Architecture and applications. BMC Bioinformatics, 10(1), 421. 10.1186/1471-2105-10-421 20003500 PMC2803857

[ece37401-bib-0012] Campana, M. G. , Corvelo, A. , Shelton, J. , Callicrate, T. E. , Bunting, K. L. , Riley‐Gillis, B. , Wos, F. , DeGrazia, J. , Jarvis, E. D. , & Fleischer, R. C. (2020). Adaptive radiation genomics of two ecologically divergent Hawai’ian honeycreepers: The ‘akiapōlā’au and the Hawai’I ‘amakihi. The Journal of Heredity, 111(1), 21–32. 10.1093/jhered/esz057 31723957

[ece37401-bib-0013] Carlton, J. M. , Adams, J. H. , Silva, J. C. , Bidwell, S. L. , Lorenzi, H. , Caler, E. , Crabtree, J. , Angiuoli, S. V. , Merino, E. F. , Amedeo, P. , Cheng, Q. , Coulson, R. M. R. , Crabb, B. S. , del Portillo, H. A. , Essien, K. , Feldblyum, T. V. , Fernandez‐Becerra, C. , Gilson, P. R. , Gueye, A. H. , … Fraser‐Liggett, C. M. (2008). Comparative genomics of the neglected human malaria parasite *Plasmodium vivax* . Nature, 455(7214), 757–763. 10.1038/nature07327 18843361 PMC2651158

[ece37401-bib-0014] Dobin, A. , Davis, C. A. , Schlesinger, F. , Drenkow, J. , Zaleski, C. , Jha, S. , Batut, P. , Chaisson, M. , & Gingeras, T. R. (2013). STAR: Ultrafast universal RNA‐seq aligner. Bioinformatics, 29(1), 15–21. 10.1093/bioinformatics/bts635 23104886 PMC3530905

[ece37401-bib-0015] Eksi, S. , Haile, Y. , Furuya, T. , Ma, L. , Su, X. , & Williamson, K. C. (2005). Identification of a subtelomeric gene family expressed during the asexual–sexual stage transition in *Plasmodium falciparum* . Molecular and Biochemical Parasitology, 143(1), 90–99. 10.1016/j.molbiopara.2005.05.010 15996767

[ece37401-bib-0016] Fleischer, R. C. , McIntosh, C. E. , & Tarr, C. L. (1998). Evolution on a volcanic conveyor belt: Using phylogeographic reconstructions and K‐Ar‐based ages of the Hawaiian Islands to estimate molecular evolutionary rates. Molecular Ecology, 7(4), 533–545. 10.1046/j.1365-294x.1998.00364.x 9628004

[ece37401-bib-0017] Garcia‐Longoria, L. , Palinauskas, V. , Ilgūnas, M. , Valkiūnas, G. , & Hellgren, O. (2020). Differential gene expression of *Plasmodium homocircumflexum* (lineage pCOLL4) across two experimentally infected passerine bird species. Genomics, 112(4), 2857–2865. 10.1016/j.ygeno.2020.03.025 32234432

[ece37401-bib-0018] Grabherr, M. G. , Haas, B. J. , Yassour, M. , Levin, J. Z. , Thompson, D. A. , Amit, I. , Adiconis, X. , Fan, L. , Raychowdhury, R. , Zeng, Q. , Chen, Z. , Mauceli, E. , Hacohen, N. , Gnirke, A. , Rhind, N. , di Palma, F. , Birren, B. W. , Nusbaum, C. , Lindblad‐Toh, K. , … Regev, A. (2011). Full‐length transcriptome assembly from RNA‐Seq data without a reference genome. Nature Biotechnology, 29(7), 644–652. 10.1038/nbt.1883 PMC3571712 21572440

[ece37401-bib-0019] Haas, B. (2020). Genome‐guided trinity de novo transcriptome assembly. https://github.com/trinityrnaseq/trinityrnaseq/wiki/Genome‐Guided‐Trinity‐Transcriptome‐Assembly

[ece37401-bib-0020] Hardy, D. (1960). Insects of Hawaii. Volume 10, Diptera: Nematocera‐Brachycera (except Dolichopodidae). University of Hawai’i Press.

[ece37401-bib-0021] Jarvi, S. I. , Atkinson, C. T. , & Fleischer, R. C. (2001). Immunogenetics and resistance to avian malaria in Hawaiian honeycreepers (Drepanidinae). Studies in Avian Biology, 22(22), 254–263.

[ece37401-bib-0022] Kim, A. , Popovici, J. , Vantaux, A. , Samreth, R. , Bin, S. , Kim, S. , Roesch, C. , Liang, L. , Davies, H. , Felgner, P. , Herrera, S. , Arévalo‐Herrera, M. , Ménard, D. , & Serre, D. (2017). Characterization of *P. vivax* blood stage transcriptomes from field isolates reveals similarities among infections and complex gene isoforms. Scientific Reports, 7(1), 7761. 10.1038/s41598-017-07275-9 28798400 PMC5552866

[ece37401-bib-0023] Kim, D. , Paggi, J. M. , Park, C. , Bennett, C. , & Salzberg, S. L. (2019). Graph‐based genome alignment and genotyping with HISAT2 and HISAT‐genotype. Nature Biotechnology, 37(8), 907–915. 10.1038/s41587-019-0201-4 PMC7605509 31375807

[ece37401-bib-0024] Lapointe, D. A. , Gaudioso‐levita, J. M. , Atkinson, C. T. , Egan, A. , & Hayes, K. (2016). Changes in the prevalence of avian disease and mosquito vectors at Hakalau Forest National Wildlife Refuge: A 14‐year perspective and assessment of future risk. Hawaii Cooperative Studies Unit Technical Report HCSU‐073, 96720, 1–57.

[ece37401-bib-0025] Lauron, E. J. , Aw Yeang, H. X. , Taffner, S. M. , & Sehgal, R. N. M. (2015). De novo assembly and transcriptome analysis of *Plasmodium gallinaceum* identifies the Rh5 interacting protein (ripr), and reveals a lack of EBL and RH gene family diversification. Malaria Journal, 14(1), 296. 10.1186/s12936-015-0814-0 26243218 PMC4524024

[ece37401-bib-0026] Lee, H. J. , Georgiadou, A. , Otto, T. D. , Levin, M. , Coin, L. J. , Conway, D. J. , & Cunnington, A. J. (2018). Transcriptomic studies of malaria: A paradigm for investigation of systemic host‐pathogen interactions. Microbiology and Molecular Biology Reviews, 82(2), e00071–17. 10.1128/MMBR.00071-17 29695497 PMC5968457

[ece37401-bib-0027] Lerner, H. R. L. , Meyer, M. , James, H. F. , Hofreiter, M. , & Fleischer, R. C. (2011). Multilocus resolution of phylogeny and timescale in the extant adaptive radiation of Hawaiian honeycreepers. Current Biology, 21(21), 1838–1844. 10.1016/j.cub.2011.09.039 22018543

[ece37401-bib-0028] Li, H. , Handsaker, B. , Wysoker, A. , Fennell, T. , Ruan, J. , Homer, N. , Marth, G. , Abecasis, G. , & Durbin, R. (2009). The sequence alignment/map format and SAMtools. Bioinformatics, 25(16), 2078–2079. 10.1093/bioinformatics/btp352 19505943 PMC2723002

[ece37401-bib-0029] Li, W. , & Godzik, A. (2006). Cd‐hit: A fast program for clustering and comparing large sets of protein or nucleotide sequences. Bioinformatics, 22(13), 1658–1659. 10.1093/bioinformatics/btl158 16731699

[ece37401-bib-0030] Love, M. I. , Huber, W. , & Anders, S. (2014). Moderated estimation of fold change and dispersion for RNA‐seq data with DESeq2. Genome Biology, 15(12), 550. 10.1186/s13059-014-0550-8 25516281 PMC4302049

[ece37401-bib-0031] Macfarlane, R. C. , & Singh, U. (2006). Identification of differentially expressed genes in virulent and nonvirulent *Entamoeba* species: Potential implications for amebic pathogenesis. Infection and Immunity, 74(1), 340–351. 10.1128/IAI.74.1.340-351.2006 16368989 PMC1346599

[ece37401-bib-0032] Oberli, A. , Slater, L. M. , Cutts, E. , Brand, F. , Mundwiler‐Pachlatko, E. , Rusch, S. , Masik, M. F. G. , Erat, M. C. , Beck, H. P. , & Vakonakis, I. (2014). A *Plasmodium falciparum* PHIST protein binds the virulence factor PfEMP1 and comigrates to knobs on the host cell surface. FASEB Journal, 28(10), 4420–4433. 10.1096/fj.14-256057 24983468 PMC4202109

[ece37401-bib-0033] R Core Team (2019). R: A language and environment for statistical computing. Vienna, Austria: R Foundation for Statistical Computing. https://www.R‐project.org/

[ece37401-bib-0034] Rovira‐Graells, N. , Gupta, A. P. , Planet, E. , Crowley, V. M. , Mok, S. , De Pouplana, L. R. , Preiser, P. R. , Bozdech, Z. , & Cortés, A. (2012). Transcriptional variation in the malaria parasite *Plasmodium falciparum* . Genome Research, 22(5), 925–938. 10.1101/gr.129692.111 22415456 PMC3337437

[ece37401-bib-0035] Roy, S. W. , & Penny, D. (2007). Widespread intron loss suggests retrotransposon activity in ancient apicomplexans. Molecular Biology and Evolution, 24(9), 1926–1933. 10.1093/molbev/msm102 17522085

[ece37401-bib-0036] Sekar, V. , Rivero, A. , Pigeault, R. , Gandon, S. , Drews, A. , Ahrén, D. , & Hellgren, O. (2020). Gene regulation of the avian malaria parasite *Plasmodium relictum*, during the different stages within the mosquito vector. bioRxiv, 204198. 10.1101/2020.07.16.204198 34023365

[ece37401-bib-0037] van Riper, C. , van Riper, S. G. , Goff, M. L. , & Laird, M. (1986). The epizootiology and ecological significance of malaria in Hawaiian land birds. Ecological Monographs, 56(4), 327–344. 10.2307/1942550

[ece37401-bib-0038] Videvall, E. (2019). Genomic advances in avian malaria research. Trends in Parasitology, 35(3), 254–266. 10.1016/j.pt.2018.12.005 30642725

[ece37401-bib-0039] Videvall, E. , Cornwallis, C. K. , Ahrén, D. , Palinauskas, V. , Valkiūnas, G. , & Hellgren, O. (2017). The transcriptome of the avian malaria parasite *Plasmodium ashfordi* displays host‐specific gene expression. Molecular Ecology, 26(11), 2939–2958. 10.1111/mec.14085 28267239

[ece37401-bib-0040] Warncke, J. D. , Vakonakis, I. , & Beck, H. (2016). *Plasmodium* helical interspersed subtelomeric (PHIST) proteins, at the center of host cell remodeling. Microbiology and Molecular Biology Reviews, 80(4), 905–927. 10.1128/MMBR.00014-16 27582258 PMC5116875

[ece37401-bib-0041] Warner, R. E. (1968). The role of introduced diseases in the extinction of the endemic Hawaiian avifauna. The Condor, 70(2), 101–120. 10.2307/1365954

[ece37401-bib-0042] Weinberg, J. , Field, J. T. , Ilgūnas, M. , Bukauskaitė, D. , Iezhova, T. , Valkiūnas, G. , & Sehgal, R. N. M. (2019). De novo transcriptome assembly and preliminary analyses of two avian malaria parasites, *Plasmodium delichoni* and *Plasmodium homocircumflexum* .Genomics, 111(6), 1815–1823. 10.1016/j.ygeno.2018.12.004 30553810 PMC6565518

[ece37401-bib-0043] Wickham, H. (2011). ggplot2. Wiley Interdisciplinary Reviews: Computational Statistics, 3(2), 180–185. 10.1002/wics.147

[ece37401-bib-0044] Woodworth, B. L. , Atkinson, C. T. , LaPointe, D. A. , Hart, P. J. , Spiegel, C. S. , Tweed, E. J. , Henneman, C. , LeBrun, J. , Denette, T. , DeMots, R. , Kozar, K. L. , Triglia, D. , Lease, D. , Gregor, A. , Smith, T. , & Duffy, D. (2005). Host population persistence in the face of introduced vector‐borne diseases: Hawaii amakihi and avian malaria. Proceedings of the National Academy of Sciences of the United States of America, 102(5), 1531–1536. 10.1073/pnas.0409454102 15668377 PMC547860

[ece37401-bib-0045] Zehtindjiev, P. , Ilieva, M. , Westerdahl, H. , Hansson, B. , Valkiunas, G. , & Bensch, S. (2008). Dynamics of parasitemia of malaria parasites in a naturally and experimentally infected migratory songbird, the great reed warbler *Acrocephalus arundinaceus* . Experimental Parasitology, 119(1), 99–110. 10.1016/j.exppara.2007.12.018 18280472

