# Supplementary material for: Transcriptome assembly and differential gene expression of the invasive avian malaria parasite Plasmodium relictum in Hawaiʻi
Source: Ecol Evol. 2021 Mar 27;11(9):4935–44. doi: 10.1002/ece3.7401 (PMC8093664; doi:10.1002/ece3.7401)
Supplement: Supplementary file 1 — Fig S1‐S2 [file ECE3-11-4935-s002.pdf]

Supplementary Figures

Transcriptome assembly and differential gene expression of the invasive avian malaria parasite *Plasmodium relictum* in Hawai‘i

Elin Videvall[1], Kristina L. Paxton[1,2], Michael G. Campana[1], Loren Cassin-Sackett[1,3], Carter T. Atkinson[4], Robert C. Fleischer[1]

[1] Center for Conservation Genomics, Smithsonian Conservation Biology Institute, National Zoological Park, Washington, DC 20008, USA

[2] Current address: Hawai‘i Cooperative Studies Unit, University of Hawai‘i at Hilo, PO Box 44, Hawai‘i National Park, HI 96718, USA

[3] Department of Biology, University of Louisiana, Lafayette, LA 70503, USA

[4] U.S. Geological Survey Pacific Island Ecosystems Research Center, Kilauea Field Station, PO Box 44, Hawai‘i National Park, HI 96718, USA

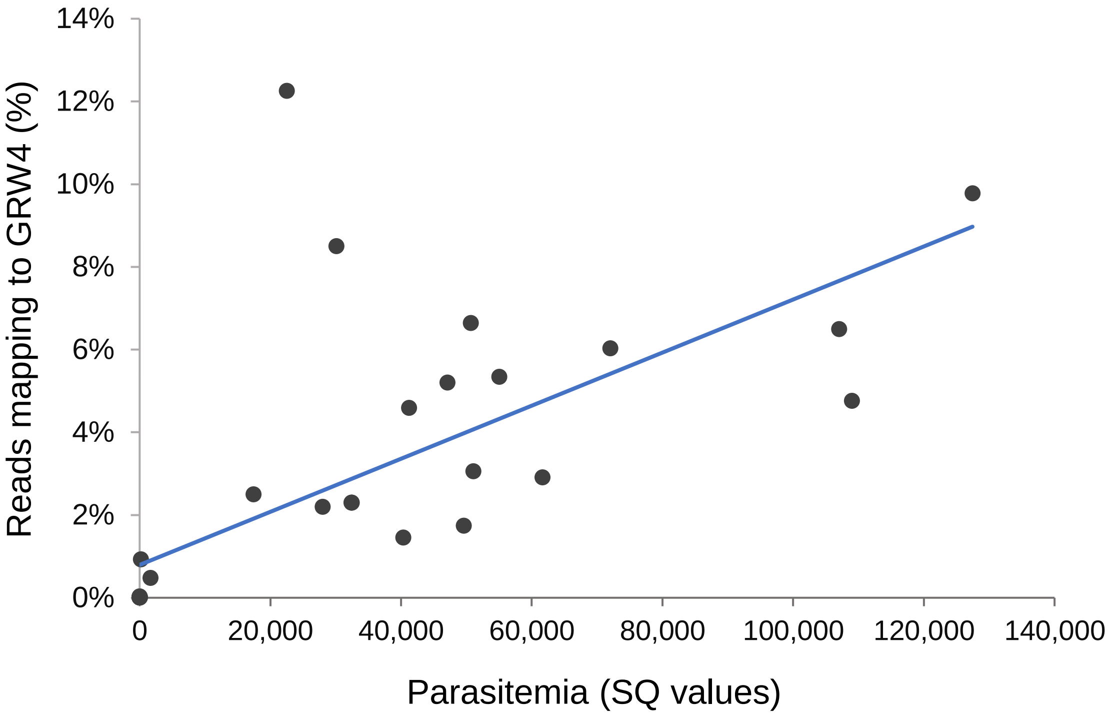

Figure S1. Parasitemia intensity (SQ values obtained from qPCR) and percent of total reads mapping to the assembled *Plasmodium relictum* GRW4 transcriptome. The blue line indicates the linear relationship (Pearson's correlation test: $t = 5.51$, $df = 32$, $p = 4.52e-06$, $r = 0.70$).

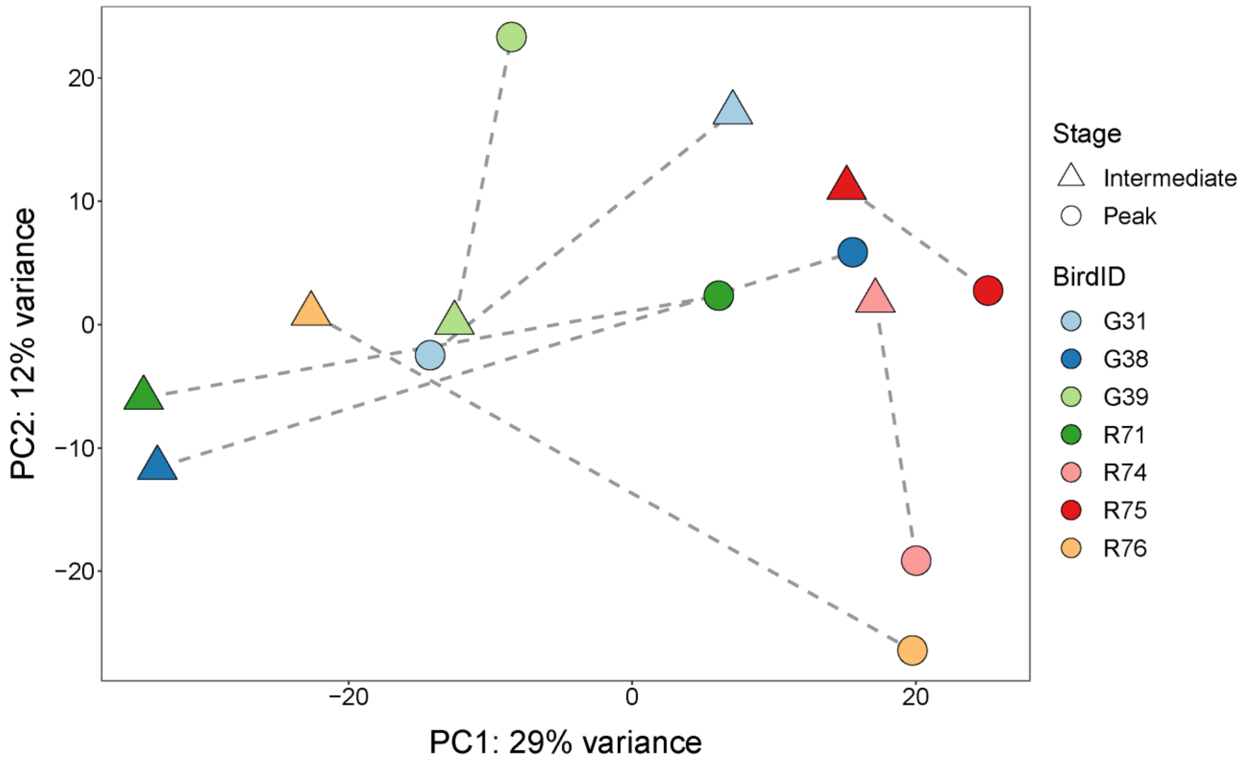

Figure S2. PCA of *Plasmodium relictum* GRW4 transcriptomes in all paired samples (host individuals with parasite transcriptional data at both intermediate and peak infection stages). Dashed lines connect parasite transcriptomes from the same host individual.
